# Biosorption of Cu^2+^ and Zn^2+^ by *Rhodotorula* sp. Kt, a Yeast Isolated from Acid Mine Drainage

**DOI:** 10.3390/ma19020418

**Published:** 2026-01-21

**Authors:** Sona Barseghyan, Narine Vardanyan, Nelli Abrahamyan, Zaruhi Melkonyan, Laura Castro, Jesús A. Muñoz, Arevik Vardanyan

**Affiliations:** 1 Institute of Microbiology, SPC “Armbiotechnology” NAS of Armenia, Yerevan 0056, Armenia; nvard@sci.am (N.V.); abrahamyan.nelly95@gmail.com (N.A.); zaruhi.melqonyan@gmail.com (Z.M.); arevik.vardanyan@asnet.am (A.V.); 2 Departamento de Ciencia de Materiales, Facultad de Química, Universidad Complutense, 28040 Madrid, Spain; lcastror@ucm.es (L.C.); jamunoz@ucm.es (J.A.M.)

**Keywords:** green technologies, biosorption, bioremediation, yeast, toxic metals

## Abstract

**Highlights:**

**What are the main findings?**

- *Rhodotorula* sp. Kt achieves 71.5% Cu^2+^ removal (pH 6 and 3 g/L), outperforming Zn^2+^ biosorption.
- Metal uptake fits pseudo-second-order kinetics (R^2^ > 0.99), indicating chemisorption.
- The Langmuir isotherm (R^2^ = 0.93) indicated monolayer adsorption with high affinity toward Cu^2+^.
- SEM-EDS confirmed surface Cu binding, supporting ICP-OES data despite a low surface signal.

**What are the implications of the main findings?**

- The yeast isolate shows promise as a cost-effective, eco-friendly biosorbent for acid mine drainage and Cu-rich waters.
- pH dependence and kinetics indicate that cell-wall functional groups drive metal uptake, guiding future optimization.
- The combined analyses provide a solid basis for improving microbial metal-removal systems.

**Abstract:**

Acid mine drainages (AMDs) enriched with toxic metals pose a significant environmental risk. Microbial bioremediation offers a sustainable and cost-effective approach for metal removal from AMD. In this study, a wild yeast isolated from the Kavart abandoned mine, identified as *Rhodotorula* sp., was evaluated for its copper (Cu^2+^) and zinc (Zn^2+^) biosorption ability. Biosorption was strongly pH-dependent. Cu^2+^ and Zn^2+^ removal was most efficient (48.1% or 10.07 mg/g and 35.7% or 6.07 mg/g, respectively) at pH 6. Increasing the biomass to 3 g/L at the same pH enhanced Cu^2+^ removal to 71.5% (26 mg/g). Biosorption kinetic analysis showed an excellent fit to the pseudo-second-order model (R^2^ > 0.99), indicating that the mechanism is chemisorption-dominated. Equilibrium data followed the Langmuir isotherm (R^2^ = 0.93), consistent with monolayer adsorption on homogeneous binding sites. SEM-EDS analysis confirmed Cu^2+^ association with the yeast surface, supporting the ICP-OES results. The results demonstrate the isolate as a promising biosorbent, particularly for Cu^2+^, and highlight its potential application in the remediation of AMD-contaminated waters.

## 1. Introduction

Toxic metal pollution is one of the most serious ecological threats caused by anthropogenic factors. The industrialization of technological progress has led to the formation of a significant amount of hazardous waste, including toxic metals (cadmium (Cd), chromium (Cr), and lead (Pb)) and metalloids (such as arsenic (As) and antimony (Sb)), which persist in ecosystems and cannot be degraded into non-toxic forms. They accumulate throughout the food chain, seriously threatening living organisms. This poses serious global health risks as these elements exceed recommended limits in water and soil, with specific concentration thresholds outlined by various regulations [1,2]. Unlike organic pollutants, these waste materials are not converted to simpler forms by chemical or biological methods but can solely be transformed into less toxic species. Removing heavy metal ions from wastewater is essential to protect public health. Industrial discharges from sectors like mining, electroplating, and energy production significantly contribute to heavy metal contamination [3,4,5]. Thus, extracting and recovering toxic metals from effluent streams is crucial for environmental protection.

Acid mine drainage (AMD), a specific type of wastewater, is a severe environmental issue associated with mining activities. AMD typically contains high concentrations of toxic metals and sulfates, and it has a low pH, posing complex challenges for remediation. Among the sources of land pollution and contamination, acid mine drainage (AMD) is a major pollutant that has contributed immensely to the deterioration of soil structure, affecting soil fertility, plant germination, growth, and proximate water bodies and leading to the gradual annihilation of terrestrial and aquatic inhabitants [2]. AMD originates from the oxidative dissolution of sulfide minerals exposed to oxygen and water, which is often accelerated by the activity of iron-oxidizing bacteria such as *Acidithiobacillus ferrooxidans*. This process produces sulfuric acid, which mobilizes toxic metals into drainage water [6].

Today, various strategies are employed to combat heavy metal pollution, including using microorganisms for bioremediation, through methods such as biosorption, bioaccumulation, bioleaching, etc., which offer a promising alternative to the existing physico-chemical methods. Microbial-based methods are often more environmentally friendly, long-term, cost-effective, and feasible and can target a wide range of contaminants under different conditions. This type of bioremediation is typically based on the local microbiota of contaminated sites [4]. Biosorption serves as a significantly more effective technique for eliminating various organic and inorganic contaminants, including toxic metals, primarily because there is a diverse range of biosorbents that are inexpensive and can easily adapt to different experimental setups [7,8,9,10]. Consequently, various microorganisms, including algae, bacteria, fungi, and yeasts, have demonstrated their capability to absorb toxic metals from liquid environments through biosorption [11,12,13]. Bacterial biosorbents bind metal ions mainly due to various functional groups, such as carboxyl and amino groups, on the cell wall. These groups create a negative surface charge that attracts metals, and factors like pH (which increases this negative charge) can further enhance binding. Additionally, modifying these groups or leveraging extracellular polymers from biofilms can improve the overall metal adsorption capacity. Negatively charged functional groups in Gram-positive (peptidoglycan, teichoic, and teichuronic acids) and Gram-negative bacteria (peptidoglycan, phospholipids, and lipopolysaccharides) primarily contribute to the cell wall’s anionic nature and metal-binding affinity [5]. After biosorption, the biomass containing the adsorbed metals can further undergo desorption with various eluents to remove metals, allowing us to reuse the biomass as a biosobent [6].

Yeasts, especially *Saccharomyces cerevisiae*, are considered effective biosorbents owing to their cell wall components, which are rich in polysaccharides and proteins capable of metal binding․ In addition to industrial yeast strains, wild yeasts may also demonstrate notable biosorptive properties, potentially due to their unique adaptations or surface characteristics [14,15,16,17]․ Furthermore, the chemical makeup of this yeast species is thoroughly documented, as is its remarkable stability over extended periods [18]. These factors have resulted in *Saccharomyces cerevisiae* being regarded as a model microorganism that possesses the capability of serving as a biosorbent for the absorption of metal ions in water, and this application is regarded as biologically safe [19].

The bioremediation potential of other non-*Saccharomyces* yeasts was studied less commonly, one of which is the genus *Rhodotorula*. *Rhodotorula* species have demonstrated bioremediation potential, as some strains can oxidize and adsorb Cu^2+^ [20], Mn^2+^ [21], and Ni^2+^ [16], enabling effective removal of heavy metals from contaminated waters.

This study aimed to evaluate the biosorption potential of a yeast strain isolated from wastewater contaminated by AMDs for copper and zinc ions under different experimental conditions, such as pH and biomass concentration. The results of this study were achieved through a systematic experimental approach designed to evaluate the biosorption potential of a yeast strain isolated from AMD-contaminated wastewater. Kinetic modeling, including pseudo-first-order, pseudo-second-order, and Elovich models, was applied to analyze the biosorption dynamics and elucidate the underlying mechanisms of metal uptake. The results may contribute to the development of effective low-cost biosorbents for heavy metal removal from contaminated waters.

## 2. Materials and Methods

### 2.1. Sampling of Acid Mine Drainage and Study of Its Physicochemical Parameters

For the isolation of yeasts resistant to high concentrations of toxic metals and acidic environments, open-pit sediment samples of the water flowing from the Kavart abandoned mine (Syunik Province, Kapan City, Armenia) (pH–2.6, t = 22 °C, altitude 960 M, N39.234708, E46.394016) were taken. Metal concentrations in water flow samples were measured using an Inductively Coupled Plasma Optical Emission Spectrometer (ICP-OES), Perkin Elmer Optima 2100 DV (Perkin Elmer Inc., Waltham, MA, USA). The elemental composition of the AMD sample is presented in Supplementary Table S1.

### 2.2. Yeast Isolation

**Figure 1:**
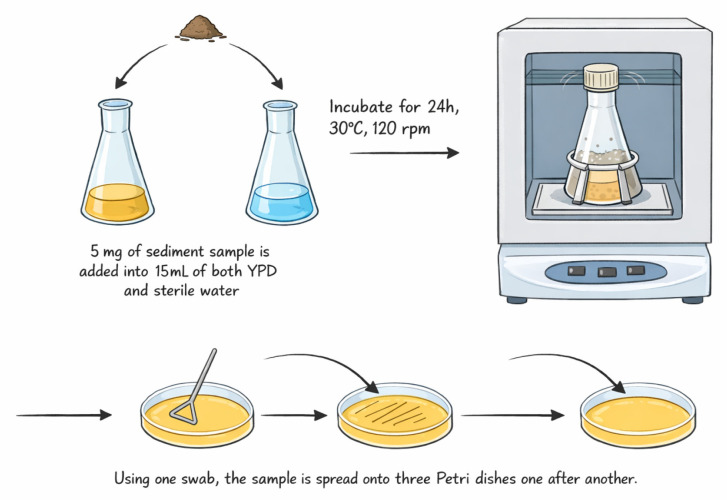
The working scheme of yeast isolation.

The sample diluted with sterile tap water (1:3), incubated overnight at 30 °C with rotation (120 rpm), was spread on YPD agar plates and incubated for 48 h (Figure 1). The YPD medium contains 20 g of dextrose, 5 g of yeast extract, 10 g of peptone, 0.2 g of chloramphenicol, and 16 g of agar in 1 L of distilled water [22,23]. Distinct colonies were aseptically picked and inoculated onto new Petri dishes twice to ensure purity, and they were transferred onto agar slants for long-term storage.

### 2.3. Phylogenetic Analysis

The total DNA of the yeast was extracted by following a protocol provided by the BioFACT Genomic DNA Prep Kit (Daejeon, Republic of Korea). Polymerase chain reaction (PCR) was performed to amplify the 18S rRNA gene region using the genomic DNA of the strain as template. The universal primers NS1 and NS6 were used. The amplified 18S rRNA gene was sequenced.

The close relative and phylogenetic affiliation of the obtained 18S rRNA sequences were determined by submitting them to the NCBI 18S ribosomal RNA GenBank database using the online NCBI BLAST tool (https://www.ncbi.nlm.nih.gov/blast). Phylogeny was inferred using the Maximum Likelihood method and the Tamura–Nei model [24]. The initial tree for the heuristic search was selected by choosing the tree with the superior log-likelihood between a Neighbor-Joining tree [25] and a Maximum Parsimony tree. The analytical procedure encompassed 21 coding nucleotide sequences using 1st, 2nd, 3rd, and non-coding positions, with 1472 positions in the final dataset. Evolutionary analyses were conducted in MEGA12 [26], utilizing up to 4 parallel computing threads.

### 2.4. Preparation of Yeast Biomass for Biosorption

A total of 100–150 mL of the liquid YPD medium was inoculated with the isolated yeast strain in 500 mL Erlenmeyer flasks and placed in a shaking incubator at 150 rpm and 35 °C for 1–2 weeks. The resulting culture liquid, with a total volume of about 650 mL, was centrifuged several times in 50 mL centrifuge tubes. The sediment was washed with distilled water, then freeze-dried. During lyophilization, the samples were first frozen in liquid nitrogen, then freeze-dried in a LyoQuest Freeze Dryer (Telstar S.A. lyophilizer; Madrid, Spain) at −82 °C under 0.072 mBar of pressure for 5 days.

### 2.5. Biosorption of Cu^2+^ and Zn^2+^ Ions at Different pH Values and Different Biomass Concentrations

The initial standard solutions (1000 mg/L) of Cu^2+^ and Zn^2+^, prepared from Cu(NO_3_)_2_ and Zn(NO_3_)_2_ (VWR, Leuven, Belgium), were stored at ambient conditions protected from light and diluted with deionized water to obtain 50 mL working solutions with a final concentration of 25 mg/L. The pH of working solutions was adjusted to pH values of 2, 4, and 6 using 0.1 M NaOH and 1 M H_2_SO_4_ and a Crison Basic 20 pH meter (Hach Lange, Barcelona, Spain). No visible precipitation was observed. The experiment was performed in duplicate at each pH value. The biomass of the lyophilized yeast was added to the metal solutions at a concentration of 1 g/L, i.e., 0.05 g per 50 mL [14,15,16,17]. Before the addition of biomass, 5 mL samples were taken. During the experiment, samples of the same volume were taken at 15, 30, 60, and 120 min after the addition of biomass. The samples were centrifuged, and metal concentrations in the supernatant were measured using ICP-OES. At the end of the experiment, the pH values of the medium were also measured.

### 2.6. Adsorption Efficiency

The adsorption efficiency of the sorbents (percentage of metal adsorption) was determined according to Formulas (1) and (2):

(1) $$R=\frac{\left(C_{i}-C_{f}\right)}{C_{f}}\times 100$$

where C_i_ is the initial concentration, C_f_ is the final concentration, and (R) is the removal percentage of the heavy metal [23].

(2) $$q=\frac{\left(C_{i}-C_{f}\right)}{m}\times V$$

where q is the metal removed per gram of yeast biomass, m is the mass of the biosorbent (g), and V is the volume of the metal solution [3,5].

### 2.7. Kinetic Modeling Methodology

To investigate the dynamics of the biosorption process and the possible mechanisms involved in heavy metal removal, several kinetic models were applied. Based on their relevance to the biosorption mechanism, the kinetic models described below were selected and evaluated. Each model is presented in its differential and linear versions. The goodness of fit of each model was evaluated based on the coefficient of determination (R^2^). Higher R^2^ values (closer to 1) indicate better correlation between the experimental data and the model, suggesting a more suitable description of the biosorption kinetics.

The pseudo-first-order (PFO) model, also known as the Lagergren first-order model, assumes that the rate of occupation of biosorption sites is proportional to the number of unoccupied sites. It is used to describe liquid–solid phase adsorption systems based on the sorbents’ absorption capacity. The differential and linear forms are given as linear (4) and differential (3) equations:

(3) $$\frac{{\mathrm{dq}}_{t}}{\mathrm{dt}}=k_{1}(q_{e}-\;q_{t})$$

(4) $$\log\left(q_{e}-q_{t}\right)=logq_{e}-\frac{k_{1}t}{2.303}$$

where $\frac{{\mathrm{dq}}_{t}}{\mathrm{dt}}$ shows the rate of biosorption at a given point in time (mg/g × min), q_t_ is the amount of metal adsorbed at a given time (mg/g), q_e_ is the equilibrium adsorption amount (when *t*→∞; mg/g), and k_1_ is the first-order adsorption rate constant (1/min).

The pseudo-second-order (PSO) model is based on the assumption that the rate-limiting step may be chemisorption involving valence forces through the sharing or exchange of electrons between the sorbent and the sorbate. The rate is proportional to the square of the number of available sites. The differential (5) and linear (6) equations are described as follows:

(5) $$\frac{dq_{t}}{dt}=k_{2}{\left(q_{e}-q_{t}\right)}^{2}$$

(6) $$\frac{t}{q_{t}}=\frac{t}{k_{2}q_{e}^{2}}+\frac{t}{q_{e}}$$

where k_2_ is the second-order adsorption rate constant (1/min).

The Elovich model is commonly used to describe the kinetics of chemisorption on heterogeneous surfaces. It assumes that the adsorption sites increase exponentially with adsorption, which is characteristic of systems with a wide distribution of activation energies. The linear (7) and differential (8) equations of the Elovich model are as follows:

(7) $$q_{t}=\frac{1}{\beta }\ln\left(\alpha \beta \right)+\frac{1}{\beta }lnt$$

(8) $$\frac{dq_{t}}{dt}={\alpha e}^{-\beta q_{t}}$$

where α is the initial adsorption rate when *q*_*t*_ = 0 (mg/g × min), while β is the deceleration coefficient related to surface saturation (1/mg) [3,5].

### 2.8. Effect of Cu^2+^ Concentration

The copper solutions for the biosorption experiments were prepared at initial concentrations of 0, 10, 25, 50, and 100 mg × L^−1^. The amount of copper adsorbed at equilibrium (qₑ, mg × g^−1^) was calculated using the difference between the initial and equilibrium concentrations. Equilibrium data were analyzed using both the Langmuir and the Freundlich isotherm models to characterize the adsorption mechanism and capacity. The equilibrium data were correlated by the Langmuir isotherm using the following formula:

(9) $$\frac{C_{e}}{q_{e}}=\frac{C_{e}}{q_{max}}+\frac{K_{L}}{q_{max}}$$

where K_L_ is the Langmuir isotherm equilibrium constant (mg/g), q_max_ is the maximum metal uptake, and *C_e_* is the equilibrium concentration of the adsorbate (mg/L).

The Freundlich isotherm was obtained using the following equation:

(10) $$q=K_{F}\times {C_{e}}^{1/n}$$

where K_F_ is a constant of the adsorption capacity, while n (dimensionless) is a constant of the adsorption intensity and nature [3,5].

### 2.9. SEM-EDS Study

A SEM-EDS study was performed using a scanning electron microscope (SEM) (JEOL JSM-6330 F (Tendo, Yamagata, Japan)) to detect metal ions, in this case copper ions, attached to the yeast cell walls. The sediment of the biomass centrifuged at the end of the biosorption process and air-dried for 2 days at 30 °C in an incubator was used as a sample, and the initial lyophilized biomass of the yeast was used as a control. For the analysis, a small piece of dry biomass was attached to adhesive tape and covered with a carbon conductive layer, which allows the detection of metal ions. The SEM was operated under high vacuum, and secondary electron detection was used to capture high-resolution images of the yeast’s cell surface morphology. Energy-dispersive X-ray spectroscopy (EDS) analysis was carried out to detect the concentration of different metals on the cell surface based on the wavelength emitted by the metal ions.

## 3. Results

### 3.1. Characterization and Selection of the Strain

After plating the experimental sample on the YPD medium and incubating it at 30 °C for about 1–2 weeks, a total of 25 distinct colonies of different morphologies were observed (on the plate with the fourth dilution), including white, cream, and reddish mucous colonies. These isolates were obtained in pure cultures using repeated streaking to ensure their homogeneity. Among the isolated yeast strains, a yeast with oval-shaped cells, forming reddish, mucous colonies, was chosen for the study. This strain, presumptively assigned to the genus *Rhodotorula*, was selected for biosorption experiments, since *Rhodotorula* spp. are frequently reported as robust biosorbents due to their cell wall composition and extracellular matrices [16,20,21]. This strain exhibited a consistent colony appearance across repeated cultivations, confirming its purity, and it was further maintained under appropriate storage conditions for subsequent experimental procedures.

### 3.2. Phylogenetic Analysis of the Isolated Yeast Strain

**Figure 2:**
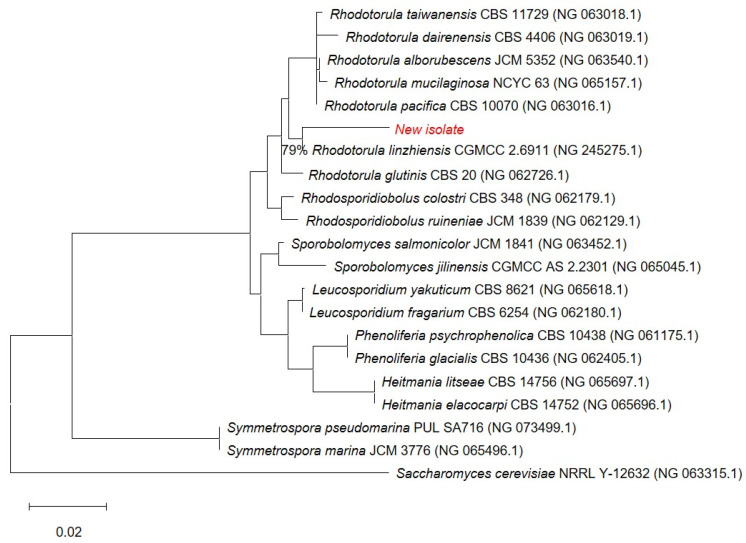
Phylogenetic position of the new isolate (in red). 0.02—scale bar. 79%—site coverage.

The novel isolate (shown in red) belongs to the *Rhodotorula* genus (Figure 2) and forms a well-supported cluster with *Rhodotorula linzhiensis* (CGMCC 2.6911), with high bootstrap support indicating a strong evolutionary link. Although the novel isolate clusters with *R. linzhiensis*, this association is based on 79% site coverage, whereas all other taxa in the alignment have full (100%) site coverage; thus, sequences with low coverage were deleted from the final figure to ensure accuracy and visual clarity. *Saccharomyces cerevisiae* was used as an outgroup to root the tree. Branch lengths correspond to the estimated number of nucleotide substitutions per site, with the scale bar representing 0.02 substitutions/site. Collectively, these results provide solid bootstrap-supported evidence that the new isolate is most closely related to *Rhodotorula linzhiensis* within the *Rhodotorula* lineage. The 18S sequence of the isolated yeast strain was submitted to the NCBI GenBank as *Rhodotorula* sp. Kt. The following accession number was assigned: PX677341.

### 3.3. Biosorption of Cu^2+^ and Zn^2+^ Ions at Different pH Values and Different Biomass Concentrations of the Isolated Yeast Strain

**Figure 3:**
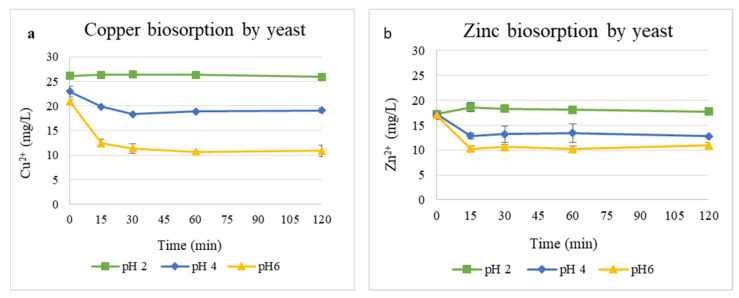
Biosorption of Cu^2+^ (**a**) and Zn^2+^ (**b**) ions at pH values of 2, 4, and 6 with 1 g/L yeast biomass.

The effect of pH on Cu^2+^ and Zn^2+^ biosorption by the yeast biomass is shown in Figure 3. Metal removal was strongly influenced by the pH of the solution. For Cu^2+^ (Figure 3a), its concentration decreased sharply from 21 to 10 mg/L at pH 6 within 30 min, while only a moderate reduction occurred at pH 4, and a negligible change was observed at pH 2. Similarly, Zn^2+^ removal (Figure 3b) from 16.99 to 10.92 mg/L was most efficient at pH 6, whereas pH 2 showed minimal removal of Zn^2+^.

At the end of the experiment, pH values of the solution were also measured to ensure that the latter remained stable during the experiment (the average value of two duplicates is presented) (Table 1). As seen from Supplementary Table S2, in the case of pH 2, the pH remained nearly constant for both Cu^2+^ and Zn^2+^ solutions throughout the 120 min incubation period. At pH 4, both metals show a notable increase in pH, especially Zn^2+^ (Cu^2+^: 4.00 to 4.46; Zn^2+^: 4.00 to 5.27). At pH 6, the trend is different: pH in the case of Cu^2+^ decreased from 6.00 to 5.24. In the case of Zn^2+^, pH increased slightly from 6.00 to 6.43 (Table S2).

Table 1 summarizes the adsorption efficiency (R, %) and the amount of metal removed per gram of yeast biomass (q, mg/g) for Cu^2+^ and Zn^2+^ at different pH values. Cu^2+^ biosorption efficiency increased markedly with pH, reaching 48.1% (10.08 mg/g) at pH 6, compared with only 0.8% (0.21 mg/g) at pH 2 and 16.6% (3.8 mg/g) at pH 4. Zn^2+^ showed its highest removal efficiency at pH 6 (35.72%, 6.07 mg/g), followed by pH 4 (25.8%, 4.45 mg/g), while adsorption was negligible at pH 2, indicating weak binding or desorption under highly acidic conditions. Overall, the results demonstrate that metal uptake increases with pH and that Cu^2+^ was adsorbed more efficiently than Zn^2+^ under the tested conditions.

**Figure 4:**
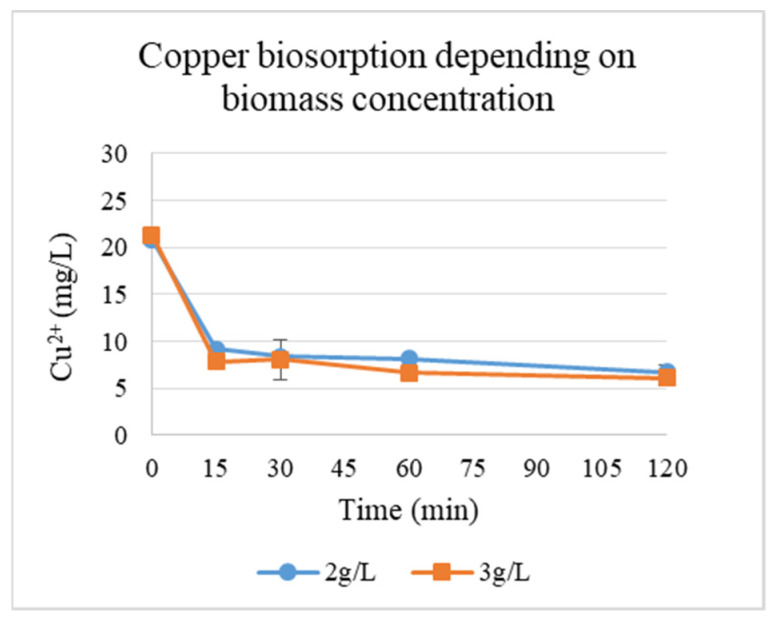
Cu^2+^ biosorption by yeasts depending on the concentration of biomass at pH 6.

Since the yeast biomass was more efficient at binding copper, the evaluation of the effect of biomass concentration (g/L) at pH 6 (the optimal pH) on biosorption of Cu ions was performed (Figure 4). The plot depicts the equilibrium adsorption of copper ions (Cu^2+^) by yeast cells as a function of biomass concentration. The results demonstrate the correlation between yeast biomass availability and metal ion uptake.

pH dynamics during Cu^2+^ biosorption by yeasts at different biomass concentrations are presented in Table 1. At a biomass concentration of 2 g/L, the pH remained stable (2.00–2.05) over 120 min, suggesting minimal proton exchange or buffering effects under acidic conditions. A slight pH increase (4.00 to 4.46) at 3 g/L biomass implies weak alkalinization, possibly due to metabolic activity or ion exchange (e.g., H^+^/Cu^2+^ competition for binding sites) [14]. Adsorption efficiency (R, %) and capacity (q, mg/g) across two biomass concentrations (2 and 3 g/L) were calculated. Thus, the concentration of the biosorbent also had a positive effect on adsorption. Up to 71.5% adsorption was recorded at 3 g/L biomass, compared to 67.7% at 2 g/L (Table 1).

### 3.4. Analysis of Kinetic Models

The kinetics of heavy metal biosorption were analyzed by applying three commonly used models: the pseudo-first-order (PFO/Lagergren), pseudo-second-order (PSO), and Elovich models. As described in the Section 2, each model was linearized according to its respective equation, and the resulting plots were used to calculate kinetic parameters and assess how well each model described the experimental data. For the pseudo-first-order model, linear plots were generated by plotting time (t, min) on the x-axis and the natural logarithm of the difference between the equilibrium adsorption capacity and the amount adsorbed at time ln(q_e_ − q_t_) on the y-axis. In the case of the pseudo-second-order model, time (t, min) was plotted on the x-axis, while t/qt was plotted on the y-axis to obtain a straight-line relationship. For the Elovich model, the natural logarithm of time (ln(t)) was plotted on the x-axis, while the amount adsorbed at time (q_t_) was plotted on the y-axis. These linearized plots enabled the determination of model-specific rate constants and provided insight into the sorption kinetics by comparing the correlation coefficients (R^2^). The coefficient of determination (R^2^) was used to assess the goodness of fit of each model to the experimental data. The R^2^ values for each kinetic model are represented below in Table 2.

The PFO model showed generally low correlation coefficients (R^2^ = 0.001 to 0.81) and extremely small rate constants (k_1_ = 0.0067 to 0.0481 min^−1^) for all systems tested, which indicates that the experimental data do not fit the model. The high R^2^ value (>0.99) observed for the PSO model provided an excellent fit to the kinetic data. In contrast, the Elovich model, which describes chemisorption on energetically heterogeneous surfaces, had a poorer fit to the experimental data (R^2^ < 0.8).

### 3.5. Effect of Cu^2+^ Concentration on Biosorption Performance

**Figure 5:**
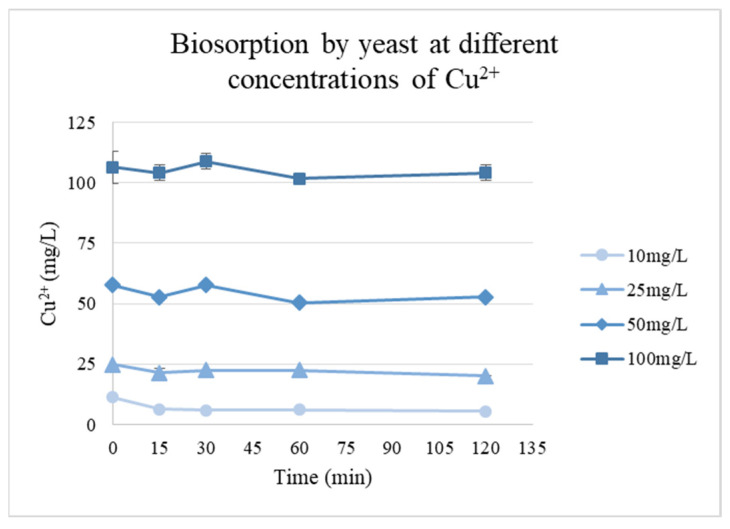
Cu^2+^ biosorption kinetics by yeast biomass at different initial metal concentrations.

The biosorption capacity of the isolated biomass evaluated using different Cu^2+^ concentrations showed that rapid uptake occurred during the first 30 min for all concentrations, followed by a slower process until an equilibrium was reached. During the experiment, the pH of the solutions with concentrations of 25 mg/L and above decreased from pH 6.0 to approximately 4–4.5, which likely contributed to the lower adsorption capacities at these higher concentrations, as the reduced pH can affect the ionization of functional groups on the biomass and the overall metal–biomass interactions (Figure 5).

The highest adsorption was observed at the lowest concentration (10 mg/L), with a q_e_ of 5.79 mg/g. For higher initial concentrations, q_e_ values decreased to 4.72, 4.93, and 2.36 mg/g for concentrations of 25, 50, and 100 mg/L, respectively.

For the Langmuir model, qₘₐₓ and K_ᴸ_ were determined from the linearized plot of Cₑ/qₑ versus Cₑ, while the Freundlich constants K_F_ and n were obtained from the linear plot of log qₑ versus log Cₑ.

**Figure 6:**
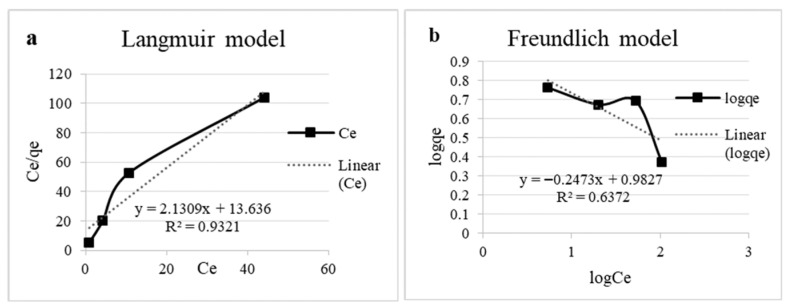
Experimental data on adsorption isotherms. (**a**) Langmuir model linear equation and (**b**) Freundlich model linear equation.

The experimental qₑ values ranged from 2.36 to 5.79 mg/g, and higher initial copper concentrations led to greater uptake despite a decrease in solution pH over time. The Langmuir (Figure 6a) model showed the best fit to the equilibrium data (R^2^ = 0.93), indicating monolayer adsorption on a homogeneous surface. The maximum adsorption capacity (q_max_) was calculated as 0.47 mg/g, with a Langmuir constant (K_L_) of 6.40 L/mg, reflecting a strong affinity of the biomass for copper ions. Conversely, the Freundlich model (Figure 6b) poorly described the adsorption process (R^2^ = 0.64), with a negative adsorption intensity parameter (*n* = −4.04), suggesting that multilayer adsorption or heterogeneous surface effects were not significant under these conditions [3,5]. The isotherm parameters are presented in Table 3.

### 3.6. SEM-EDS Results

**Figure 7:**
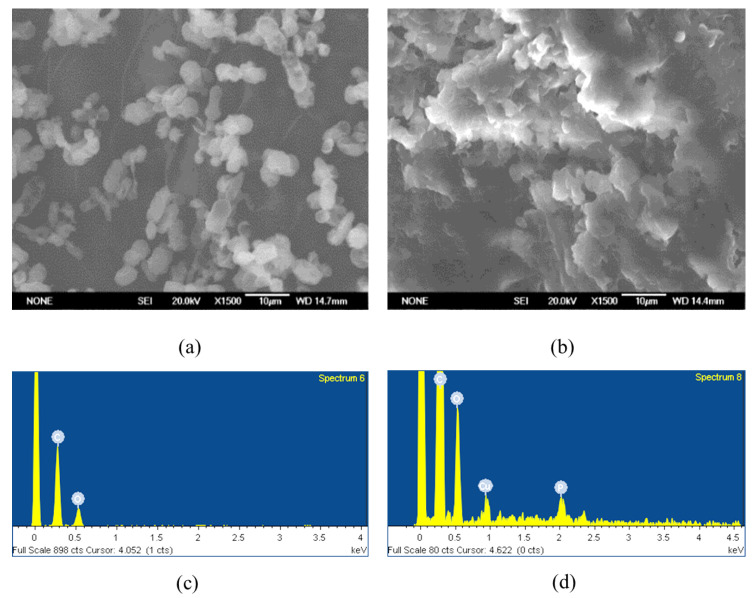
Yeast cells before treatment with heavy metal solutions: in a lyophilized state (**a**) after thermostatic drying and after biosorption of copper with 1 g/L biomass (**b**) at pH 6 under a scanning electron microscope. The amount of elements detected on the cell surface by EDS analysis before (**c**) and after biosorption of copper (**d**) with 1 g/L biomass at pH 6.

The SEM-EDS study result indicates that due to the high compactness and aggregation of the dried sample, obtaining detailed high-resolution images of the entire structure was challenging. Nevertheless, individual yeast cells could still be distinguished within the dense matrix (Figure 7a,b), allowing localized EDS measurements to be carried out. The EDS analysis confirmed the presence of copper ions on the yeast cell surface (Figure 7c,d). Taken together, the SEM-EDS results provide qualitative confirmation that copper is associated with the biomass.

## 4. Discussion

### 4.1. The Influence of pH and Biomass on the Adsorbtion Kinetics

The most significant decrease in the case of both Zn^2+^ and Cu^2+^ occurred within the first 15–30 min, indicating rapid binding of metal ions to the yeast surface, followed by equilibrium stabilization [14]. Across all tested conditions, the interaction between metal-containing solutions resulted in distinct pH dynamics. At pH 2, the stability of pH for both metal solutions suggested that proton exchange between the biomass and the medium was minimal, likely due to saturation of active sites because of high proton concentrations. At pH 4, both metals induced a noticeable increase in pH, especially for Zn^2+^, indicating that metal uptake is accompanied by proton consumption. In contrast, at pH 6, distinct behaviors were evident: Cu^2+^ induced a slight decrease in pH, whereas Zn^2+^ caused a minor alkaline shift. pH decrease in the presence of Cu^2+^ indicates proton release associated with metal binding, consistent with high-affinity interactions involving carboxyl and phosphate functional groups. Conversely, the slight pH increase observed in Zn^2+^ solutions may reflect weaker ion-exchange mechanisms or partial precipitation processes [27].

These results indicate that biosorption was accompanied by slight pH variations, particularly at higher initial pH values. pH changes highlight metal-specific mechanisms of biosorption and underscore the strong influence of initial pH on metal–biomass interactions [28].

The consistently higher uptake of Cu^2+^ compared to Zn^2+^ suggests a stronger affinity of yeast biomass toward copper, possibly due to different binding constants or preferential interactions with specific functional groups, such as thiol and carboxyl groups.

The strong dependence of metal removal on pH reflects the protonation state of cell-wall functional groups involved in metal binding. These results showed a clear dependence on the acidity of the solution for copper absorption, which is consistent with literature data, according to which the biosorption of metals increases in a neutral medium due to the deprotonation of active groups of the cell wall (e.g., -COOH → -COO^−^) [29]. The evaluation of biomass concentration at the optimal pH further supports this trend: increasing the amount of biomass increases available binding sites, resulting in enhanced Cu^2+^ uptake until saturation is reached.

The enhanced adsorption at 3 g/L compared with 2 g/L reflects the expected increase in total binding sites. In terms of pH dynamics, pH remained nearly constant, indicating minimal proton exchange, which is consistent with the increase in available binding sites.

Together, the results indicate that copper biosorption is jointly influenced by pH and biomass concentration. A similar effect has been shown in various studies [30,31,32]. Below are the results of biosorption studies by various yeast species, which were used for comparative analysis with the obtained results (Table 4) [14,20,33]. The biosorption performance of the isolated yeast strain was comparable against several previously reported values for *Saccharomyces cerevisiae*, indicating its high potential for heavy metal removal under the tested conditions.

### 4.2. Kinetic Studies

In terms of kinetic modeling, the poor fit to the PFO model suggests that Cu^2+^ and Zn^2+^ biosorption by the yeast is not determined by physical adsorption or mass transfer of ions to the surface of the sorbent. When chemisorption or more intricate binding mechanisms predominate, studies of microbial- and plant-based biosorbents often report poor fits of the PFO model. For instance, the PFO model has frequently been found to be less effective in biosorption studies on yeast and other microbial biomass [14,34]. The PSO model, in contrast, shows an excellent fit, which suggests that the rate-limiting step in the biosorption of Cu^2+^ and Zn^2+^ ions may be chemical, rather than being an effect of simple diffusion or physical adsorption. This interpretation is consistent with numerous recent experimental studies in which PSO kinetics best described heavy metal uptake by microbial biomass, indicating chemisorption-dominated processes [35,36]. The relatively weaker performance of the Elovich model suggests that the biosorbent surface contains relatively homogeneous active sites with similar adsorption energies [14,37]. Overall, the adsorption mechanism can be connected with a uniform distribution of reactive sites, where chemical interactions, rather than surface heterogeneity or physisorption processes, occur. This conclusion is consistent with recent experimental reviews and comparative studies of microbial biosorbents that highlight PSO (chemisorption) as the controlling pathway for Cu^2+^ and Zn^2+^ uptake.

### 4.3. Isotherm Modeling

In terms of isotherms, the rapid uptake during the first 30 min and subsequent slower phase, decreased efficiency at higher initial metal concentrations, likely caused by the pH drop, indicate that the biosorbent has a limited number of active sites, which become progressively saturated at higher Cu^2+^ concentrations, reducing the adsorption efficiency under low-pH conditions. Other yeast-based systems have shown similar saturation effects, indicating the finite nature of biosorbent binding capacity [37]. The superior fit to the Langmuir model suggests that copper biosorption by the biomass is primarily a monolayer process (Table 3). The decrease in pH during the experiments may have influenced adsorption at higher copper concentrations, but the overall adsorption behavior remained consistent with a single-layer binding model.

Overall, yeast biosorption of Cu^2+^ and Zn^2+^ is strongly influenced by pH, biomass concentration, and metal-specific interactions. The process is primarily chemisorption-driven, with monolayer binding at homogeneous active sites, and significant intracellular accumulation may occur beyond what surface analyses reveal.

## 5. Conclusions

This study highlights the potential of a wild yeast strain isolated from AMD as a biosorbent for heavy metal removal. The results demonstrate a pH-dependent and biomass concentration-dependent biosorption process of copper (Cu^2+^), with the highest efficiency (71.5%) at pH 6 and a biomass concentration of 3 g/L. In contrast, zinc (Zn^2+^) biosorption was less effective, with a maximum removal efficiency of 35.7% under similar conditions.

Kinetic modeling indicated that the pseudo-second-order model best describes the biosorption process of both Cu^2+^ and Zn^2+^, suggesting that chemisorption takes place, which slows down the process. SEM-EDS analysis, despite the limited biomass density and surface sensitivity, supported the accumulation of copper on yeast surfaces, complementing the quantitative results obtained via ICP-OES. The Langmuir and Freundlich isotherm analyses indicated that the biosorption process generally involves monolayer adsorption, while heterogeneous surface interactions are rarely involved.

Overall, the findings suggest that the yeast strain has significant biosorptive capabilities, specifically for copper, under optimized conditions. This supports its potential application as a cost-effective and environmentally friendly biosorbent for the treatment of AMD and other metal-contaminated effluents. Future research should aim to elucidate the molecular mechanisms of metal binding, including the characterization of extracellular polymeric substances (EPSs) produced by the yeast; assess desorption efficiency and reusability; and validate the biosorbent’s performance in real wastewater systems. Additional experiments using both living and inactivated (dead or lyophilized) biomass will allow a comparison of metal removal efficiencies and help distinguish passive biosorption from metabolism-dependent bioaccumulation. Multimetallic systems will also be investigated to assess the competitive interactions among different ions, which typically occur in natural wastewaters such as acid mine drainage (AMD). Complementary analyses, such as Fourier Transform Infrared Spectroscopy (FTIR) and Transmission Electron Microscopy (TEM), can be applied to identify functional groups involved in metal binding and to observe intracellular metal accumulation, thus verifying the underlying removal mechanisms.

## Figures and Tables

**Table 1:** Adsorption efficiency of the sorbent at different biomass concentrations (g/L).

Metal	Biomass Concentration, g/L	pH	Removal Efficiency, R (%)	Adsorption Capacity, q (mg/g)
Cu^2+^	1	2	0.78	0.20
		4	16.57	3.80
		6	48.09	10.07
	2	6	67.68	28.17
	3		71.55	30.51
Zn^2+^	1	2	2.75	0.47
		4	25.80	4.45
		6	35.72	6.07

**Table 2:** Key parameters for the kinetic models of each condition.

Kinetic Model	Parameters	Cu^2+^					Zn^2+^		
		Biosorbent Biomass, g/L							
		1			2	3	1		
		pH 2	pH 4	pH 6	pH 6		pH 2	pH 4	pH 6
PFO (Lagergren)	k_1_	0.0134	0.0067	0.0116	0.0347	0.0481	0.0096	0.0075	0.0090
	R^2^	0.7688	0.2813	0.2813	0.6658	0.8034	0.9853	0.2813	0.2813
PSO	q_e_	−0.2040	3.8329	10.2145	7.0872	5.1361	4.8222	4.3995	6.0864
	k_2_	79.0157	0.3617	0.0807	0.0471	0.0889	−0.4675	0.1034	0.0999
	R^2^	0.9956	0.9949	0.9991	0.9966	0.9986	0.9488	0.9915	0.9972
Elovich	α	−2.9 × 10^−5^	207,689.0723	5164.2148	1528.2767	21,999.4442	166.2986	−4.45 × 10^−124^	−1.71 × 10^−15^
	β	−172.4138	4.5005	1.3053	1.8359	3.1476	2.4067	−66.2251	−4.4425
	R^2^	0.0308	0.1034	0.7673	0.9876	0.5747	0.9876	0.0018	0.3889

**Table 3:** Parameters for adsorption isotherms.

Langmuir	
R^2^	0.9321
q_max_ (mg/g)	0.4693
K_L_ (L/mg)	6.3990
Freundlich	
R_2_	0.6372
K_F_ ((mg/g) × (L/mmol)1/*n*)	9.6101
*n*	−4.0438

**Table 4:** Adsorption of toxic metals by various yeast species.

Microorganism	Metal	Metal Concentration	Biomass	pH	Adsorption %	Reference
*Saccharomyces cerevisiae*	Zn (II),	25 mg/L	Dead, 4 g/L	5	81.98%,	[14]
	Cu (II)				75.41%	
*Pichia pastoris*	Cu (II)	100 mg/L	Wet, 0.025 g/L	6	41.1%	[33]
*Rhodotorula mucilaginosa*	Cu (II)	50 mg/L	Live, -	-	~35%	[20]

## Data Availability

The original contributions presented in this study are included in the article/supplementary material. Further inquiries can be directed to the corresponding author.

## Supplementary Materials

The following supporting information can be downloaded at https://www.mdpi.com/article/10.3390/ma19020418/s1. Table S1: The physicochemical parameters and chemical composition of the samples; Table S2: pH values at the beginning and end of the biosorption experiment.

## Abbreviations

The following abbreviations are used in this manuscript:

AMD	Acid mine drainage
ICP-OES	Inductively coupled plasma optical emission spectroscopy
YPD	Yeast–peptone–dextrose
PFO	Pseudo-first-order
PSO	Pseudo-second-order
SEM	Scanning electron microscope
EDS	Energy-dispersive X-ray spectroscopy
