# Supplementary material for: Biosorption of Cu2+ and Zn2+ by Rhodotorula sp. Kt, a Yeast Isolated from Acid Mine Drainage
Source: Materials (Basel). 2026 Jan 21;19(2):418. doi: 10.3390/ma19020418 (PMC12843149; doi:10.3390/ma19020418)
Supplement: Supplementary file 1 [file materials-19-00418-s001.zip › materials-4075681-supplementary Table S1.pdf]

Table S1: The physicochemical parameters and chemical composition of the samples

Sampling site	Kavart open-pit sediment (June, 2024)
Sampling location (GPS)	N39.234708, E46.394016
pH/Eh	2.6/620.1
Temperature (°C)	12
Chemical elements (λ, nm)*	mg/L
Al (396.152)	143.72
Ca (317.933)	361.94
Co (238.892)	0.03
Cr (283.892)	1.38
Cu (327.395)	18.04
Fe (238.204)	674.66
K (766.491)	0.96
Mg (279.553)	133.6
Mn (259.372)	6.07
Na (589.592)	0.8
Ni (230.299)	0.38
Pb (405.781)	0.06
Se (203.985)	0.02
Sr (407.771)	0.01
Zn (202.548)	0.66

*λ is the characteristic absorption wavelength of the elements
