# Supplementary material for: Biosorption of Cu2+ and Zn2+ by Rhodotorula sp. Kt, a Yeast Isolated from Acid Mine Drainage
Source: Materials (Basel). 2026 Jan 21;19(2):418. doi: 10.3390/ma19020418 (PMC12843149; doi:10.3390/ma19020418)
Supplement: Supplementary file 1 [file materials-19-00418-s001.zip › materials-4075681-supplementaryTable S2.pdf]

Table S2. pH values at the beginning and end of the biosorption experiment.

Biomass dosage of *Rhodotorula* sp. Kt, g/L	1						2		3	
pH	2		4		6		6		6	
Time, min	0	120	0	120	0	120	0	120	0	120
pH change for Cu^{2+}	2.00	2.05	4.00	4.46	6.00	5.24	6.00	5.46	6.0	5.49
pH change for Zn^{2+}	2.00	2.06	4.00	5.27	6.00	6.43	-	-	-	-
